# Medical and Philosophical Intelligence

**Published:** 1815-07

**Authors:** 


### MEDICAL AND PHILOSOPHICAL INTELLIGENCE.

*ROYAL Society.*—On Thursday, the 4th of May, a paper by Sir Humphrey Davy was read, on the action of acids on hyper-oxymuriate of potash. In consequence of the discovery of a new acid by Gay-Lussac by treating hyper-oxymuriate of barytes with sulphur, Sir H. Davy was induced to examine more carefully than had hitherto been done the action of acids on the hyper-oxymuriate of potash. When sulphuric acid is poured upon this salt in a wine-glass, very little effervescence takes place, but the acid gradually acquires an orange colour, and a dense yellow vapour of a peculiar and not disagreeable smell floats on the surface. These phenomena led the author to believe, that the substance extricated from the salt is held in solution by the acid. After various unsuccessful attempts to obtain this substance in a separate state, he, at last, succeeded by the following method. About 60 grains of the salt are triturated with a little sulphuric acid, just sufficient to convert them into a very solid paste. This is put into a retort, which is heated by means of hot water. The water must never be allowed to become boiling hot, for fear of explosion. The heat drives off the new gas, which may be received over mercury. This new gas has a much more intense colour than euchlorine. It does not act on mercury. Water absorbs more of it than of euchlorine. Its taste is astringent. It destroys vegetable blues without reddening. When phosphorus is introduced into it, an explosion takes place. When heat is applied, the gas explodes with more violence, and producing more light, than euchlorine. When thus exploded, two measures of it are converted into nearly three measures, which consist of a mixture of one measure chlorine and two measures oxygen. Hence, it is composed of one atom chlorine, and four atoms oxygen. It is not unlikely, that euchlorine is a simple mixture of three measures of chlorine and two measures of this new gas; but the point cannot be determined till it be known whether Dutch foil will burn burn in such a mixture as it does in euchlorine. This experiment the author could not try, because at Rome, where he then was, he could procure no Dutch foil fit for his purpose. The same gas is disengaged from hyper-oxymuriate of potash by nitric acid, and with still greater facility; but it is always mixed with one-fifth of its bulk of oxygen gas. Sir H. Davy conceives, that the chloric acid of Gay-Lussac contains hydrogen, and owes its acid properties to the presence of this principle.

At the same meeting of the society, a paper by Dr. Philips was read, giving an account of the remains of a fœtus found within the abdomen of a child. The child was aged two years and a half, and had a swelling in the belly, which had been considered as dropsical, and aperient medicines had been administered. The swelling did not diminish; and the child at last was obliged to be kept always in a recumbent posture. Dr. Philips, on examining the abdomen, found a circumscribed swelling on the left side, which, at first, he considered as an enlarged spleen, but afterwards laid aside that opinion, without being able to form any one in the least satisfactory. The child died on the fourth day after having been seen by Dr. Philips. On opening the abdomen, the intestines were all found in a sound state, except the liver, which was indurated. The tumor consisted of a large mass of matter not connected particularly with any of the intestines, weighing eight or ten pounds, and inclosed in a very vascular bag. On cutting into it, some serous liquid oozed out. The dissection, from the situation of the medical men, was necessarily hurried and imperfect; but unequivocal traces of a fœtus were found, particularly the bones of the tibia and talus, and some others, which were found adhering together, and covered with muscle.

On the 25th of May, a paper by Dr. Parry was read, on the nature and cause of the pulse. Dr. P. took a review of the different theories which have been proposed to account for the phænomenon of pulsation, observing that the greater part of physiologists had contented themselves with the opinion of Haller, that pulsation was occasioned by the diastole and systole of the heart. His view, however, of the question is much simpler; on examining different arteries where they were exposed to no obstruction or pressure, he found that they had no pulse: by pressing the finger on an artery over a soft part of the body, which yielded sufficiently to the pressure, no pulse was manifested; but, whenever an artery was pressed over a solid part, then a pulse was immediately found. He repeated these operations several times, and uniformly found the same effects. Hence he concludes, that the pulse is nothing more than the re-action or impetus of the blood to maintain its regular motion. The arteries appear only as canals through which the blood flows in a uniform and continuous current; diminish the diameter of the canals, and a pulse is immediately perceived. At every junction of a vein with an artery, the internal diameter of the latter is diminished, and hence a pulse always appears. This Dr. P. thinks fully adequate to account for all the modifications of the pulse.

Part of a very elaborate paper was read, detailing numerous experiments on malic acid, analysis of several vegetables which contain this acid, and on the crystallization of malat of lead. Gooseberries, and many other culinary vegetables contain considerable quantities of malic acid; but on discovering that malat of lead sometimes formed crystals, and at others continued in a thick mucilaginous state, the author was induced to vary his experiments to ascertain the cause of this apparent anomaly, when he discovered that it was owing to the presence of another acid. This acid also existing in vegetables, when combined with the malic, the solutions of lead in it then assume a crystalline character. This fact is of great importance, as malat of lead has been deemed to be one of our most delicate tests or re-agents.

---

*Linnæan Society.*—On Tuesday, May 2, a paper by George Montague, Esq. on the ardea nigra, or black stork, was read. This bird was shot in England.

On Wednesday, the 24th of May, the anniversary of the Society, the following office-bearers were elected for the ensuing year:—

*President*, Sir James Edward Smith, M.D.—*Treasurer*, Thomas Marsham, Esq.—*Secretary*, Alexander Macleay, Esq.—*Under Secretary*, Mr. Richard Taylor.

There were retained of the old Council:—The President.—The Lord Bishop of Carlisle.—Aylmer Bourke Lambert, Esq.—William Elford Leach, M.D.—Alexander Macleay, Esq.—Thomas Marsham, Esq.—William George Maton, M.D.—Daniel Moore, Esq. F.R.S.—Joseph Sabine, Esq. F.R.S.—Thomas Smith, Esq.

The five following fellows were elected into the council:—Thomas, Marquis of Bath.—William Kent, Esq.—Rev. Thomas Rackett.—Thomas Thomson, M.D. F.R.S.—John Walker, Esq.

Since last anniversary, the society has lost nine fellows and five foreign members by death; and eleven new fellows have been elected into the society.

---

*Medical Society of the University of Dublin.*—This society was instituted during the preceding winter. It consists of medical practitioners and students; the former are divided into foreign honorary, corresponding, and resident-honorary members; the latter, into privileged and ordinary members. The objects of the society are two-fold: first, to collect original information on all branches of medical science, with a view to publication; secondly, to improve the junior members of the society, by their writing dissertations on medical subjects and publicly defending them.

At the conclusion of the session, a donation of twenty guineas was presented to the society, by the professor of anatomy in the university, for the purpose of rewarding the best essay that shall be sent to the society before the 1st of April, 1816, on the following question: "What differences exist between venous and arterial blood, with respect to chemical composition and vital properties?"

perties?" The answers to this question are to be supported by new and decisive experiments. They may be written in Latin, German, French, or English, and are to be addressed to the Secretary of the Medical Society, Trinity College, Dublin, accompanied by a sealed packet, containing the author's name and place of residence.

---

At a general meeting of the students of the Anatomical Class, Trinity College, it was resolved, that the following address be presented to Dr. Macartney :—

"SIR.—The class who have had the great advantage of attending your lectures during the present session, cannot separate from you at the approaching recess, without testifying to you their warm sense of gratitude for the instruction they have derived from you.

"It would be presumptuous in them to dwell upon your acknowledged talents and professional abilities, but they may be permitted to bear testimony to your laborious and unremitted exertions for the improvement of medical science, and particularly that most important branch of it, Morbid Anatomy, the proof of which is evinced, and will be long preserved, in the very valuable collection which your activity and acute discernment have enabled you to form, in a shorter space of time than probably has ever been effected by any individual.

"Allow them to offer you their best thanks, and grateful acknowledgments, for your zealous and unwearied attention to every subject connected with their studies, and for the affectionate and friendly interest you have always taken in their improvement.

"*Friday, 5th of May*, 1815."

To which Dr. Macartney returned the following answer :—

"GENTLEMEN.—I beg leave to return you my very sincere thanks for the expressions of partiality and attachment contained in your address, this day presented to me.

"It affords me peculiar satisfaction to find that you set a just value on Morbid Anatomy, a species of research by which, alone, the physician can arrive at any certainty in judging of the nature of most diseases submitted to his care.

"The time and labour that I have devoted to the obtaining and preserving specimens of Morbid Structure, were only consistent with my opinion of the importance of the objects, and my duty to you. But be assured, I esteem no efforts of mine too great which are rewarded with the affection of my pupils.

"Allow me, gentlemen, to offer you my warmest wishes for your prosperity and happiness.

"*Leeson-street, May* 6, 1815." "JAMES MACARTNEY."

---

*Small-Pox Infection.*—The Board of the National Vaccine Establishment did, much to its credit, in April last, prosecute and convict, in the Court of King's Bench, a woman at Paddington, for exposing her child, after inoculation, in the streets, when covered vered with small-pox pustules; by which misconduct, she infected eleven persons; eight of whom died, and another lost the use of one eye.

She was indicted upon an old law, which declares, the exposure of a person in a public place with any infectious disease which endangers the lives of others, to be a criminal act, and punishable by law. As this was the first indictment for such an offence, the punishment was mitigated to three months imprisonment: the object of the prosecution being, in this instance, rather to manifest, that persons so offending are amenable to existing laws; and to make an example to deter the ignorant or evil-disposed from similar practices.

The following is the trial of Mr. GILBERT BURNET, Apothecary, of Great Mary-le-bone-Street, for causing children to be exposed after inoculation for the small-pox.

KING'S BENCH, JUNE 7.—THE KING, *v.* GILBERT BURNET.

The defendant, who is an apothecary residing in Great Mary-le-bone-street, had been indicted for causing children whom he had inoculated for the small-pox to be exposed improperly in the public streets and highways, to the imminent danger of communicating the infection to others of his Majesty's subjects. The defendant, it appeared, had suffered judgment to go by default, and was now brought up to receive the judgment of the court.

Affidavits were read, proving that the defendant had been in the practice of inoculating a considerable number of children, and that he had them daily taken to him by their parents, whilst the complaint was full out upon them, and whilst the disorder was in that stage in which it was likely the infection would be communicated to persons who had not previously had the disease.

Mr. OWEN addressed the court in arrest of judgment, and submitted that the defendant had been guilty of no indictable offence, since the inoculation of children, the offence with which the defendant was charged, was no offence at common law; nor was the inoculating of persons in his own house, by the defendant, a subject of indictment. If it was so the system pursued at the Small-Pox Hospital was equally a subject which called for the interference of the legal authorities of the country.

Lord ELLENBOROUGH observed, that that establishment to which the learned counsel alluded, was one of the finest in the country, and did not come within the scope of the observations applicable to the present case. When the Small-Pox Hospital was erected, care was taken to select a spot distant from any neighbourhood. And one of the regulations of that establishment was, that persons labouring under this infectious malady, should never be exposed in public. Since its erection, however, a neighbourhood had collected about it, and, should it now become the subject of indictment, it must share the fate of all others, and all its fine buildings and lofty walls must fall.—With respect to the motion for arrest of judgment, there was nothing in it. The defendant was not indicted for inoculating his patients, but for exposing them unlawfully, when they were in a state likely to communicate infection, and thereby endangering the safety of his Majesty's subjects generally.

Affidavits of Sarah Milburne, of Mary-le-bone-Mews, and of Sarah Shute, were put in and read. They stated, that in June last, they had children inoculated by the defendant, to whom they were attracted by his public advertisement. That during the progress of this disorder, they repeatedly applied to the defendant to visit their children at home, which he refused to do, and in consequence, their parents were obliged to take them through the street, and that on their different visits they had seen numerous children there, and met them going to and returning from the defendant's house, with the disease full out upon them. The children of both deponents died.

The affidavit of the defendant stated, that he had suffered judgment to go by default in consequence of the derangement of his affairs, although he had a good defence to the action; that so far from advising persons to expose, he had uniformly cautioned all parties not to expose themselves while labouring under the disease; that he was subject to a spitting of blood, and that imprisonment for any length of time would prove fatal to him.

The Attorney-General had not now to contend that inoculation was illegal, or that any man was not at liberty to inoculate his whole family, or to let them remain till Providence should think fit to visit them with the small-pox, in the natural way, if he thought proper; all he meant to contend was, that he should do this with proper caution, and in such a manner as not to endanger the safety of others. All he had to contend for, and he was convinced the court would, by their judgment that day, prove that he was right, was, that no man was to be at liberty to make a trade of poisoning the air, and infecting the health of the public as he should think proper, and as the defendant had done.

Mr. Owen, in mitigation, contended, that the prosecutors had dealt unfairly by the defendant, in selecting out of the numerous patients of the defendant two cases where the children had died. No person felt more for this circumstance than he did; but, with equal justice might a barrister be arraigned for making an unsuccessful defence at the Old Bailey. This client was poor in the extreme, almost as poor as the starved apothecary in Romeo and Juliet.

Mr. Justice Le Blanc, in passing sentence, said, the court was not called on to give any opinion as to the merits of the different systems of inoculation. It had always been illegal to inoculate in an infectious way, as this defendant had done. To deter others from committing the like offence, the sentence of the court was, that the defendant be committed to the custody of the marshal of that court for six calendar months.

---

The *Journal de Medicine* for February last, has an ingenious paper paper on rheumatism of the heart. It contains, however, nothing very different from those remarks published by Mr. Dundas, in the Medico-Chirurgical Transactions. At the conclusion is a note by the compiler, on the rheumatism of the œsophagus. "Troubled for years with a rheumatic pain, which, at different times, assailed different parts of the body, he at length felt it, for three days, in the muscles surrounding the breast; when, on a sudden, he perceived, about the middle of the œsophagus, a sense of constriction which rendered deglutition very painful. The similarity of these last affections to those of the muscles, made him particularly attentive to each. They both arose about the same time, and ceased about the same time, after a profuse perspiration."

---

We have lately witnessed a very interesting case at the Institution for the Cure of Cataract, in Windmill-street. The patient, William Baldock, a boy, eleven years old, was born with cataracts; that in the left eye was the common opake cataract, particularly dense; the right was the *black* cataract, which we do not recollect to have seen described before in a congenital case. Mr. Stevenson has performed the operation in both eyes with complete success, and the boy has returned to his friends to Mayfield, Sussex, capable of distinguishing, with accuracy, the smallest dots on paper.

---

The age of charms, it seems, is not yet ended. A young man had been subject to epilepsy about two years; the disease followed a complaint which was regarded as hydrocephalus internus, for which he had been cupped and blistered several times, besides a long course of medicines. When the epileptic fits first attacked him, his bodily strength was much reduced, but he was free from the original affection of the head. The medicines which, under different practitioners, he took for the epilepsy, not proving beneficial, he was induced to try the efficacy of an amulet. This consisted in placing, on one of his fingers, a ring, formed of silver, contributed by twelve young women. He has, since the experiment, been free from fits for a month, although he generally had several in the course of a week; and now wears his ring in full confidence of having no further return of his complaints, which rendered him incapable of pursuing his profession.

---

The Journal Desprachschen Halkund contains an interesting case of instant death, by Prussic acid, the particulars of which we shall give in our next.

---

*Surgeons' Bill.*—We are authorized to state, that provisions have been inserted in the Surgeons' Bill respecting Midwifery, &c. which are satisfactory to the London Committee of Associated General Practitioners.

---

Dr. Adams has published, at the request of the Right Honourable Sir Joseph Banks, a Philosophical Treatise on the Hereditary Peculiarities of the Human Race; with Notes illustrative of the Subject, particularly Gout, Scrofula, and Madness. The second edition, with an Appendix on the Goîtres and Cretins of the Alps and Pyrenees. The Appendix may be had separate.

METEO-

### METEOROLOGICAL REGISTER.

*From May the 25th, to June the 25th,* 1815.

Kept by C. BLUNT, Philosophical Instrument Maker, No. 38, Tavistock-Street, Covent-Garden.

| Moon. | Day. | Wind. | Barometrical Pressure. Max. | Min. | Mean. | Temperature. Max. | Min. | Mean. | |
|---|---|---|---|---|---|---|---|---|---|
| | 26 | W | 30·16 | 30·12 | 30·145 | 66 | 52 | 59 | Fair |
| | 27 | W | 30·16 | 30·11 | 30 085 | 66 | 51 | 58·5 | Fair |
| | 28 | W | 29 90 | 29 90 | 29·90 | 67 | 53 | 60 | Fair |
| | 29 | W | 29·84 | 29·78 | 29·757 | 65 | 50 | 57·5 | Fair |
| | 30 | NW | 29·87 | 29 87 | 29·87 | 65 | 49 | 57 | Fair |
| ◑ | 31 | NW | 29·87 | 29·81 | 29 84 | 64 | 49 | 56 5 | Rain |
| | 1 | W | 29·89 | 29·79 | 29·885 | 66 | 55 | 60·5 | Fair |
| | 2 | W by S | 30·04 | 30·04 | 30 04 | 68 | 57 | 62·5 | Fair |
| | 3 | W | 29·97 | 29·89 | 29 917 | 67 | 56 | 61·5 | Fair |
| | 4 | W | 29·76 | 29·76 | 29 76 | 67 | 55 | 61 | Fair |
| | 5 | NW | 29·63 | 29·54 | 29·577 | 66 | 54 | 60 | Rain |
| | 6 | NW | 29·50 | 29·44 | 29·467 | 68 | 56 | 62 | Rain |
| ● | 7 | NW | 29·57 | 29·57 | 29 57 | 67 | 55 | 61 | Fair |
| | 8 | NNW | 29·80 | 29 72 | 29·747 | 65 | 51 | 58 | Rain |
| | 9 | N | 29·85 | 29·84 | 29·845 | 65 | 48 | 56·5 | Fair |
| | 10 | N | 29·84 | 29·80 | 29·827 | 66 | 47 | 56·5 | Rain |
| | 11 | NW | 29·79 | 29·79 | 29·79 | 67 | 49 | 58 | Fair |
| | 12 | W | 29 79 | 29·71 | 29·742 | 65 | 52 | 57 | Rain |
| | 13 | W | 29 54 | 29·54 | 29·54 | 67 | 54 | 60 5 | Rain |
| ◐ | 14 | W | 29·38 | 29·34 | 29·35 | 69 | 52 | 60·5 | Fair |
| | 15 | WSW | 29·76 | 29·54 | 29·652 | 70 | 53 | 61·5 | Fair |
| | 16 | S | 29 86 | 29·78 | 29·817 | 70 | 53 | 61·5 | Fair |
| | 17 | S | 29 59 | 29·57 | 29 83 | 71 | 54 | 62·5 | Fair |
| | 18 | S | 29 66 | 29 66 | 29·66 | 70 | 51 | 60·5 | Rain |
| | 19 | S | 29·74 | 29·60 | 29·687 | 70 | 50 | 60 | Rain |
| | 20 | SW | 29 66 | 29·66 | 29 66 | 69 | 50 | 59 5 | Fair |
| ○ | 21 | W | 29·86 | 29 75 | 29·82 | 69 | 51 | 60 | Fair |
| | 22 | NW | 29 85 | 29 83 | 29 835 | 70 | 53 | 61·5 | Rain, Aurora Borealis at Night |
| | 23 | NW | 30· | 29·91 | 29 955 | 68 | 50 | 59 | Fair |
| | 24 | NNW | 30 03 | 29 96 | 29·992 | 68 | 49 | 59·5 | Fair |
| | 25 | NNW | 30 02 | 30·02 | 30·02 | 68 | 49 | 58·5 | Fair |

RESULTS.

| | | | | | |
|---|---|---|---|---|---|
| Mean barometrical pressure of the month | | 29·7929 | Mean temperature of the month | | 56·387 deg. |
| Maximum 30·16 | wind at | W | Maximum 71, | wind at | S |
| Minimum 29·34 | ——— | W | Minimum 47, | ——— | N |

*Scale exhibiting the prevailing Winds during the Month.*

| N | NE | E | SE | S | SW | W | NW |
|---|---|---|---|---|---|---|---|
| 5 | 0 | 0 | 0 | 4 | 1 | 13 | 8 |

| | Mean barometrical pressure | Mean temperature |
|---|---|---|
| From the last quarter on the 31st May, to the new moon on the 7th of June | 29·745 | 61·214 |
| ——— new moon on the 7th, to the first quarter on the 14th | 29·691 | 58·142 |
| ——— first quarter on the 14th, to the new moon on the 21st | 29·752 | 60·785 |

REPORT

### REPORT OF DISEASES.

Since the last report, very few cases of fever have occurred, and those were not of a malignant character. Rheumatism has been very frequent, both in the acute and chronic form. In some cases of acute rheumatism, the pain and other symptoms were only removed by copious and repeated bleedings, succeeded by purgatives and diaphoretics. In general, the lancet is too much neglected in this disease. In the chronic form, calomel combined with opium in the proportion of one grain of each, every night, with large doses of tinct. guaiac. ammon. during the day, usually procured relief. Sometimes friction is requisite, and, in many instances, a small dose of emetic tartar may be added to the calomel and opium with good effect. It need hardly be observed, that keeping the bowels open in both forms of the disease, always assisted the other remedies.

Pulmonic disease has not been rare.

In a case of peripneumony, bleeding was not practised in the beginning of the complaint, and the patient was nearly lost; but ultimately recovered, by the physician who was called in, immediately directing blood to be taken away, in as large a quantity as the patient could bear.

Several cases of phthisis and hæmoptysis have occurred. In Dispensary-practice the physician is often enabled to see those complaints at an early period; and, where the patient does not resist the plan of treatment proposed, the disease is frequently cured. A vegetable diet, avoiding wine, beer, &c., repeated bleedings according to the symptoms, purgatives, anodynes, with squils, digitalis, &c. have proved the most useful remedies. With all these, tranquillity of mind, and exercise, when used so gentle as not materially to increase the circulation, considerably assist in the restoration of health. If these do not seem to succeed, change of air, or even of climate, is the last chance; but this is often too long deferred. But in these cases, the patients themselves most frequently accelerate the progress and increase the violence of the disorder, by acting contrary to their physician's instructions: it requires as much art to combat prejudice, as skill to ascertain and devise the right mode of treating a disease.

